# Global, regional, and national burden of preterm birth 1990 to 2023, with projections of prevalence to 2050

**DOI:** 10.3389/fpubh.2026.1889106

**Published:** 2026-07-30

**Authors:** Qi Huang, Yaling Zhou, Jiaming Li, Chengjun Song, Lizhong Du, Chao Yu, Yanping Xu

**Affiliations:** 1 Neonatal Intensive Care Unit, Children's Hospital, Zhejiang University School of Medicine, National Clinical Research Center for Children and Adolescents' Health and Diseases, Hangzhou, China; 2 Neonatology, Children's Hospital, Zhejiang University School of Medicine, Hangzhou, China; 3 College of Life Sciences, Zhejiang University, Hangzhou, China

**Keywords:** forecasting, global burden, low- and middle-income countries, neonatal mortality, preterm birth

## Abstract

**Background:**

Preterm birth, the leading cause of under-five mortality, resulted in approximately 0.6 million neonatal deaths in 2023 and significant lifelong morbidity for survivors, including neurodevelopmental and respiratory impairments. This study aims to provide an updated assessment of age-standardized rates (ASR) in preterm birth prevalence, incidence, mortality, and disability-adjusted life-years (DALYs) from 1990 to 2023. Comprehensive, updated estimates are vital to guide resource allocation to neonatal intensive care units (NICUs), target maternal health interventions, and inform national prevention policies.

**Methods:**

Using data from the Global Burden of Disease 2023, we analyzed the global, regional, and national burden of preterm birth from 1990 to 2023, extracted estimating prevalence, incidence, deaths, DALYs, and age-standardized rates. Trends were assessed via estimated annual percentage change (EAPC), with data stratified by age, sex, location, Socio-demographic Index (SDI). Analyses were performed using R software (Version 4.5.0).

**Results:**

In 2023, there were 125.5 million global cases of preterm birth, representing a 53.9% increase since 1990. The age-standardized prevalence rate (ASPR) increased slightly (EAPC: 0.40; 95% CI: 0.38–0.42) and age-standardized incidence rate (ASIR) decreasing (EAPC: −0.33; 95% CI: −0.40 to −0.27), suggesting the tremendous success of global neonatal healthcare over the past few decades. Despite these improvements, 623,303 deaths and 70.2 million DALYs were attributed to preterm birth in 2023, with age-standardized death rate (ASDR) and DALY rates decreasing (EAPCs: −2.39 and −2.03, respectively). The highest burden was observed in South Asia and Sub-Saharan Africa, which collectively exceeding 11 per 100,000 population of ASDRs particularly in low- and low-middle SDI regions. Looking ahead, projections based on historical trends and sociodemographic forecasts suggest that while absolute prevalence is expected to rise slightly by 2050, mortality and DALY rates will continue to decline.

**Conclusions:**

The global burden of preterm birth presents a dual challenge: while age-standardized deaths and DALY rates are declining, the absolute number of prevalence continues to rise, driven by population growth. To meet Sustainable Development Goals requires a dual-pronged strategy: strengthening neonatal intensive care in high-burden regions and advancing maternal health equity through preventative interventions.

## Introduction

Preterm birth, the WHO defined as delivery before 37 completed weeks of gestation since the first day of the women's last menstrual period, is the leading cause of death among children under five. Despite global initiatives like the Every Newborn Action Plan and the Sustainable Development Goals (SDGs) aiming to reduce under-five mortality, the absolute burden of preterm birth has remained high, with the greatest burden concentrated in regions such as South Asia and Sub-Saharan Africa (1, 2).

The impact is particularly severe in low- and middle-income countries, where health system limitations, such as a shortage of experienced obstetricians and neonatologists, weak referral systems, and limited access to essential interventions like antenatal corticosteroids and neonatal intensive care, exacerbate risks associated with preterm delivery (3, 4). While previous analyses, such as the Global Burden of Diseases (GBD) 2019, neonatal disorders, including those resulting from preterm birth, are among the top causes of disability-adjusted life-years (DALYs) in children under 5 years of age (5, 6). However, previous studies have often lacked global comparability over recent years, stratification by Socio-demographic Index (SDI), and crucial forward-looking projections, all of which limit their utility for contemporary policy-making. Therefore, this study aims to provide a comprehensive, up-to-date analysis.

Preterm birth is a multifactorial condition with complex etiologies, including medical risks (e.g., maternal infections, hypertensive disorders), socioeconomic determinants, behavioral factors, emerging environmental exposures and genetic predispositions (7–9). Although antenatal corticosteroids, improved obstetric care, and neonatal intensive care have shown promise in specific settings, the global implementation of these strategies remains uneven (10, 11). Preterm birth is not only associated with high rates of death but also leads to a wide range of short- and long-term complications, including respiratory distress syndrome (RDS), and neonatal sepsis, while long-term consequences for survivors often involve significant neurodevelopmental impairment and chronic lung disease (12). Addressing the burden of preterm birth requires comparable, and up-to-date data to identify populations at greatest risk and to monitor progress toward global targets.

To address these gaps, this study sought to determine the global, regional, and national trends in the burden of preterm birth (prevalence, incidence, deaths, and DALYs) from 1990 to 2023, and to project its prevalence to 2050. We hypothesized that despite progress in reducing age-standardized deaths rates, the absolute burden of preterm birth has increased, driven by population growth and persistent disparities. Using the updated GBD 2023, we provide a comprehensive analysis disaggregated by age, location, and Socio-demographic Index (SDI) to inform public health strategies and support evidence-based decision-making to reduce the burden of preterm birth globally.

## Methods

### Study design and data collection

This study is based on data from the GBD 2023 study, sourced from the Global Health Data Exchange (GHDx) (https://vizhub.healthdata.org/gbd-results/). Collaborators from around the world contribute to compiling data, organized by age and gender, at the national and regional levels of the SDI using uniform procedures. we focused on eight principal metrics: directly extracted prevalence, incidence, mortality, DALYs, and modeled estimates of age-standardized prevalence rate (ASPR), age-standardized incidence rate (ASIR), age-standardized death rate (ASDR) and age-standardized DALY rates associated with preterm birth. The data, spanning from 1990 to 2023, was sourced from the GHDx and includes annual statistics across location, sex, age, risk factors, and year. Our analysis covered 204 countries and territories, grouped into 21 GBD regions based on geographic proximity and further classified into five categories according to the socio-demographic index (SDI).

### Case definition

In the GBD study, preterm birth is defined as birth before 37 completed weeks of gestation and is categorized under the broader cause group of neonatal disorders. It involves a systematic data harmonization process where GBD collaborators synthesize information from diverse sources. Case identification was based on ICD-10 codes P07.2–P07.3, as well as corresponding ICD-9 codes.

### Categorization of locations

We categorized locations worldwide using the SDI. SDI is a summary measure that identifies where countries or other geographical areas sit on the spectrum of development. Expressed on a scale of 0–1, SDI is a composite average of the ranking of the income per capita, average educational attainment, and fertility rates of all areas in the GBD study. Locations are then categorized within SDI quintiles, termed low, low-middle, middle, high-middle, and high. SDI is specific by country and year and, thus, the categorization of a specific country can change over time (13). This stratification is vital for interpreting disparities in the preterm birth burden across different levels of socioeconomic development.

### Data analysis

To assess the trends in age-standardized rates of preterm birth, prevalence, incidence, mortality and DALYs, the study utilized the Estimated Annual Percentage Change (EAPC) for the period from 1990 to 2023. The calculation of EAPCs was based on a regression model that characterizes the pattern of age-standardized rates during a specified period (14). The EAPC and its 95% confidence interval (CI) were derived from a log-linear regression model fitted to the natural logarithm of the rates (y = α+βx +ε, where y = ln(ASR) and x = calendar year). The EAPC was then calculated as 100 × (e^∧^β-1). An ASR is deemed to have an increasing trend if both the EAPC and the lower bound of its 95% CI are positive. In contrast, if both the EAPC and the upper bound of its 95% CI are negative, the ASR is considered to have a decreasing trend. If neither condition is satisfied, the age-standardized rate is regarded as stable. All analyses and visualizations were carried out using the R statistical computing software (Version 4.5.0), with key packages including dplyr for data management and ggplot2 for plotting.

## Results

### Global level

**Figure 1:**
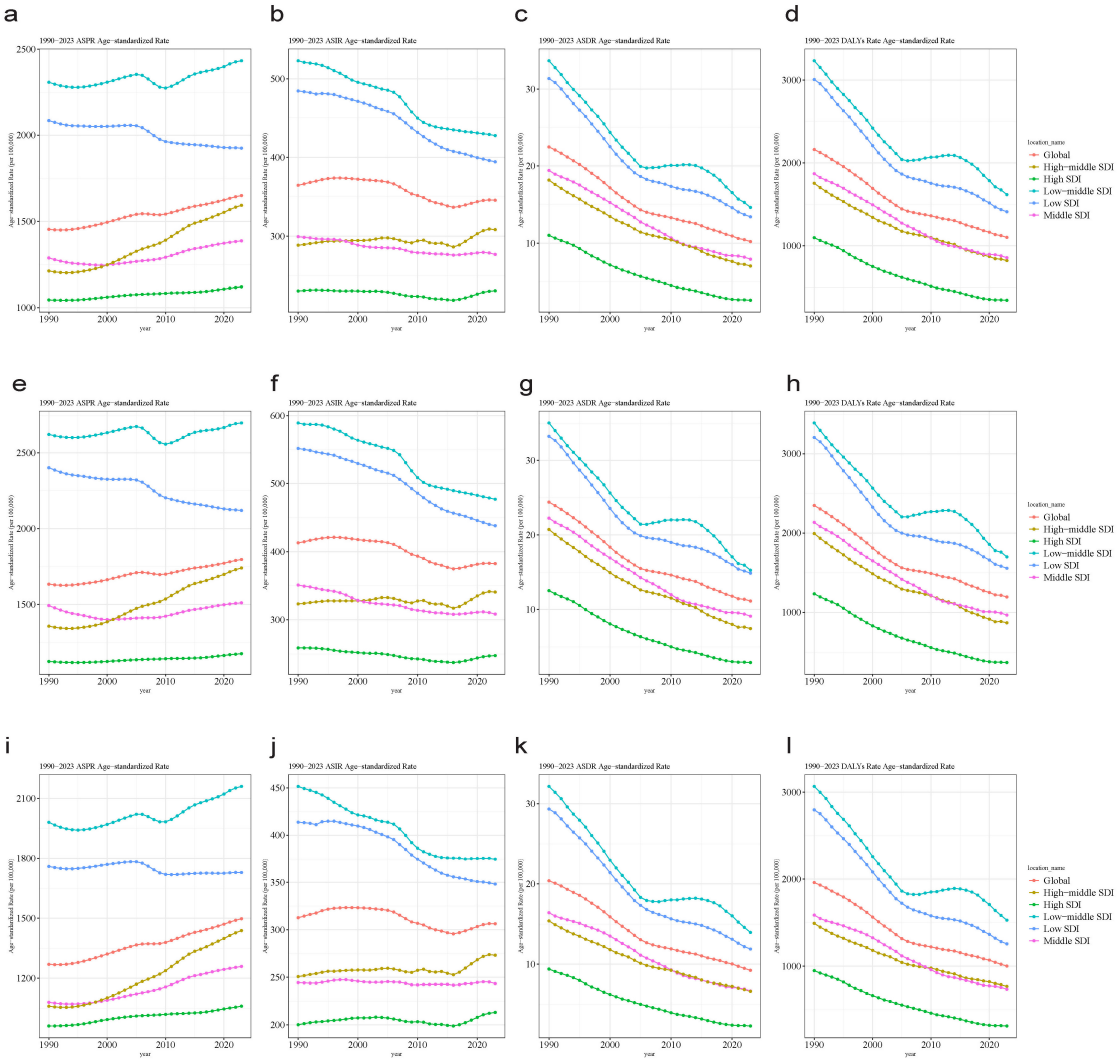
Trends in preterm birth prevalence, incidence, deaths and disability-adjusted life-years from 1990 to 2023**. (a)** Both-ASPR per 100,000; **(b)** Both-ASIR per 100,000; **(c)** Both-ASDR per 100,000; **(d)** Both-DALYs per 100,000; **(e)** Male-ASPR per 100,000; **(f)** Male-ASIR per 100,000; **(g)** Male-ASDR per 100,000; **(h)** Male-DALYs per 100,000; **(i)** Female-ASPR per 100,000; **(j)** Female-ASIR per 100,000; **(k)** Female-ASDR per 100,000; **(l)** Female -DALYs per 100,000.

In 2023, the global burden of preterm birth remained substantial, with an estimated 125.5 million cases (95% uncertainty interval [UI]: 108.9–142.5 million), representing a 53.9% increase compared to 1990. Despite this marked rise in the absolute number of cases, the ASPR showed only a modest increase, rising from 1,453.5 per 100,000 population (95% UI: 1,240.7–1,637.9) in 1990 to 1,649.5 per 100,000 (95% UI: 1,439.0–1,862.7) in 2023, reflecting population growth rather than worsening risk. The EAPC for ASPR was 0.40 (95% confidence interval [CI]: 0.38 to 0.42), indicating a relatively stable trend over time (Table 1, Figure 1). In contrast, the global incidence of preterm birth in 2023 was 21.1 million cases (95% UI: 20.9–21.2 million), reflecting a 8.55% decrease from 1990. The ASIR declined from 364.6 per 100,000 (95% UI: 356.2–361.8) in 1990 to 345.6 per 100,000 (95% UI: 345.6–351.3) in 2023. Correspondingly, the EAPC for ASIR was −0.33 (95% CI: −0.40 to −0.27), suggesting a consistent reduction in the incidence rate over the study period (Table 1, Figure 1). In terms of mortality, preterm birth accounted for an estimated 623,303 deaths globally in 2023 (95% UI: 509,687–735,006), with an ASDR of 10.2 per 100,000 (95% UI: 10.1–14.2). The EAPC in ASDR was −2.39 (95% CI: −2.5 to −2.28), indicating a notable decline in mortality associated with preterm birth, a trend likely attributable to significant advances in neonatal intensive care (Table 1). This presents a dual burden of disease: while the age-standardized DALY rate also consistently decreased (EAPC: −2.03; 95% CI: −2.14 to −1.92), the absolute number of DALYs remained immense at 70.2 million in 2023 (95% UI: 60.6–81.4 million), underscoring the vast, ongoing impact of long-term disability from preterm birth (Table 1, Figure 1).

**Table 1:** Global and regional trends in neonatal preterm birth burden: prevalence, incidence, mortality, and disability-adjusted life years (1990-2023).

Location	1990		2023		EAPC_95%CI
	Number	ASR	Number	ASR	
Prevalence					
Global	81,510,440.7 (69,966,719.3–91,649,643.9)	1,453.5 (1,240.7–1,637.9)	125,503,885.2 (108,883,399.5–142,503,904.9)	1,649.5 (1,439–1,862.7)	0.4 (0.38 to 0.42)
High SDI	20,490,507.8 (17,466,761–23,247,338)	1,044.7 (897.1–1,179)	24,406,266.8 (21,045,772.6–28,084,930)	1,120.3 (986.6–1,267.3)	0.21 (0.2 to 0.22)
High-middle SDI	14,351,462.7 (12,329,695.5–16,306,055.7)	1,212.2 (1,038–1,380.3)	23,258,964.7 (20,291,460.8–26,314,955.4)	1,592.9 (1,398.1–1,790.2)	0.97 (0.92 to 1.02)
Middle SDI	8,527,145.4 (7,384,513.2–9,770,712)	1,287.5 (1,105.4–1,486.5)	12,913,936.7 (11,020,826.7–14,693,696)	1,386.7 (1,189–1,571.3)	0.32 (0.26 to 0.38)
Low-middle SDI	18,306,446 (15,338,449.9–21,853,287.4)	2,308.6 (1,902.5–2,799.3)	28,783,180.2 (24,903,737.6–33,247,989.5)	2,433.4 (2,109.7–2,804.5)	0.15 (0.11 to 0.2)
Low SDI	19,769,712.8 (16,725,431.4–23,052,062.9)	2,084.6 (1,716.5–2,482.6)	36,057,934.6 (31,158,503–41,314,954.7)	1,924.7 (1,643.5–2,236.3)	−0.27 (-0.3 to−0.24)
Andean Latin America	494,316.4 (408,254.4–623,658.5)	1,165.2 (934.8–1,502.2)	648,618.7 (549,309.1–782,966.1)	1,014.7 (865–1,217.8)	−0.43 (-0.54 to−0.32)
Australasia	239,876.6 (205,215.4–295,125.6)	1,231.2 (1,058.4–1,503.8)	357,696.8 (292,265.8–443,341.5)	1,230.1 (1,025.8–1,494.6)	−0.02 (-0.06 to 0.01)
Caribbean	649,783.5 (536,332.4–804,753.3)	1,736.3 (1,422.4–2,169.4)	807,203.1 (666,060.5–1,012,702.3)	1,763.2 (1,468.7–2,201.5)	0.09 (0.02 to 0.16)
Central Asia	625,285.1 (537,108–731,147.5)	818.1 (694.1–968.7)	812,797.5 (695,708.1–950,949.7)	809.6 (692.7–948.4)	−0.07 (-0.15 to 0.01)
Central Europe	1,114,742 (933,211–1,290,883.2)	951.6 (806.9–1,090.7)	1,081,432.6 (889,983.6–1,254,486.8)	1,090.8 (924.1–1,239.8)	0.48 (0.46 to 0.51)
Central Latin America	1,647,296.3 (1,421,175.4–1,891,951.3)	897.8 (763.7–1,041.4)	2,629,069.2 (2,257,582.5–3,038,976.6)	1,082.1 (937.6–1,240.4)	0.76 (0.7 to 0.82)
Central Sub-Saharan Africa	729,985.7 (565,672.7–1,056,523.4)	1,031.9 (737.9–1,623.8)	1,619,906.1 (1,272,400.8–2,109,743.9)	948.8 (715.8–1,282.4)	−0.34 (-0.4 to−0.29)
East Asia	9,910,601.4 (8,562,601.5–11,185,600.2)	820.2 (710.1–924.7)	8,168,944.5 (7,079,439.7–9,434,498.1)	651.7 (578.6–736)	−0.69 (-0.82 to−0.57)
Eastern Europe	1,491,248.8 (1,260,086–1,702,522.3)	715.5 (612.6–809.8)	1,463,075.6 (1,243,295.5–1,691,895.2)	840.9 (735.1–951.5)	0.61 (0.56 to 0.66)
Eastern Sub-Saharan Africa	3,266,999.4 (2,735,461–3,987,813.5)	1,371.8 (1,096.5–1,750.6)	7,041,350.1 (6,161,220.9–8,113,414)	1,363.8 (1,175.5–1,599.3)	0 (-0.01 to 0.01)
High-income Asia Pacific	1,178,518.3 (996,384.9–1,386,784.6)	757.8 (651–882.6)	1,309,459.9 (1,102,631.3–1,532,944.4)	866.7 (755.4–992.8)	0.49 (0.42 to 0.55)
High-income North America	4,115,043.6 (3,472,593.3–4,760,364.1)	1,531.6 (1,300.9–1,762.3)	5,260,325 (4,482,167.2–6,164,878)	1,576.4 (1,366.2–1,817.7)	−0.06 (-0.18 to 0.05)
North Africa and Middle East	5,953,588.6 (5,070,163.2–6,776,609.7)	1,554.9 (1,299–1,802.3)	10,339,239.1 (8,864,711.9–11,899,644.3)	1,639.4 (1,408.4–1,881.6)	0.21 (0.15 to 0.28)
Oceania	124,005.2 (100,956.5–165,506.4)	1,625 (1,274.6–2,255.8)	270,415.8 (214,941.2–376,992.1)	1,651.1 (1,275.2–2,359.7)	0.02 (0.02 to 0.03)
South Asia	34,385,988.1 (28,823,564.8–40,186,289.9)	2,885.6 (2,392.1–3,403.6)	58,236,308.6 (50,031,372.5–67,674,106.2)	3,125 (2,694.5–3,620.7)	0.24 (0.21 to 0.28)
Southeast Asia	6,221,725.3 (5,333,643.2–7,047,162.5)	1,242.5 (1,054.8–1,417.2)	8,198,432.3 (7,040,431.5–9,376,311)	1,215.1 (1,052.1–1,380.2)	0.02 (-0.02 to 0.06)
Southern Latin America	493,420.6 (407,156.1–606,731.8)	983.8 (810.6–1,211.4)	800,721.2 (676,566.8–982,904.7)	1,273.4 (1,096.5–1,535.7)	0.94 (0.9 to 0.98)
Southern Sub-Saharan Africa	731,280.6 (623,721.3–866,434.1)	1,231.5 (1,028.2–1,492.4)	1,240,927.6 (1,029,452.8–1,468,278.2)	1,406.3 (1,166.6–1,662.8)	0.45 (0.43 to 0.47)
Tropical Latin America	1,664,484.6 (1,415,490.8–1,967,974.2)	1,052.9 (890.5–1,251.2)	2,982,714.1 (2,584,239.9–3,441,712.2)	1,462.3 (1,279.5–1,670.9)	1.25 (1.12 to 1.38)
Western Europe	3,534,972.5 (2,997,510.2–4,075,236.8)	1,014.5 (877.9–1,153.6)	4,414,162.9 (3,753,349.9–5,119,498.3)	1,145.8 (997–1,303.3)	0.42 (0.39 to 0.45)
Western Sub-Saharan Africa	2,937,278 (2,568,226.9–3,377,465.2)	1,206.6 (1,020.8–1,426.1)	7,821,084.5 (6,756,623–8,979,932.3)	1,284.1 (1,082.8–1,504.6)	0.2 (0.19 to 0.22)
Incidence					
Global	23,042,084.3 (22,861,762–23,226,577.9)	364.6 (361.7–367.5)	21,072,032.8 (20,918,951.6–21,219,453.8)	345.6 (343.1–348)	−0.33 (-0.4 to−0.27)
High SDI	3,666,364.9 (3,620,683.2–3,712,242.4)	230.7 (227.8–233.5)	2,397,865.7 (2,371,367.2–2,423,718.2)	231 (228.4–233.5)	−0.12 (-0.18 to−0.07)
High-middle SDI	3,586,746.2 (3,535,271.1–3,635,790.3)	288.6 (284.4–292.5)	2,559,746.2 (2,528,835.7–2,589,947.6)	308.3 (304.6–312)	0.08 (0.03 to 0.14)
Middle SDI	2,447,437.9 (2,397,336.3–2,495,787)	299.4 (293.3–305.3)	2,135,016.7 (2,095,456.1–2,176,870.4)	276.9 (271.8–282.4)	−0.26 (-0.29 to−0.23)
Low-middle SDI	5,654,348.9 (5,554,009.9–5,749,666.8)	522.8 (513.6–531.6)	4,468,257.9 (4,352,612–4,578,852.7)	427.6 (416.5–438.2)	−0.72 (-0.77 to−0.67)
Low SDI	7,671,655.3 (7,553,012.5–7,795,750.8)	484.5 (477–492.4)	9,495,605.5 (9,381,022.8–9,605,470.1)	394.4 (389.6–398.9)	−0.73 (-0.78 to−0.67)
Andean Latin America	147,878.8 (140,789.8–155,531.3)	259.8 (247.4–273.3)	120,488.5 (113,399–126,962.8)	219.9 (206.9–231.7)	−0.54 (-0.67 to−0.41)
Australasia	28,177.1 (26,672.3–29,631.3)	183.5 (173.7–193)	35,626.9 (32,359–38,327.9)	206.8 (187.8–222.4)	0.37 (0.33 to 0.42)
Caribbean	155,803.5 (149,749.5–160,851.3)	362.2 (348.1–373.9)	147,609.6 (141,857.2–153,858)	388.2 (373.1–404.6)	0.19 (0.13 to 0.24)
Central Asia	181,150.2 (173,852.9–188,093.2)	189.6 (182–196.9)	181,196.8 (172,199.4–189,893)	181.8 (172.8–190.5)	−0.22 (-0.24 to−0.19)
Central Europe	170,224.4 (165,400.6–174,843.1)	204.4 (198.6–210)	102,295.9 (99,673.7–104,975.6)	219.8 (214.2–225.6)	0.23 (0.21 to 0.26)
Central Latin America	509,048.8 (495,867.8–520,965.7)	213.8 (208.3–218.8)	427,392.8 (415,512.5–438,959.2)	238.5 (231.9–245)	0.44 (0.4 to 0.49)
Central Sub-Saharan Africa	381,019.6 (360,162.3–405,368.8)	305.8 (289.1–325.4)	600,149.2 (569,559.5–629,944.6)	254.6 (241.6–267.2)	−0.64 (-0.68 to−0.6)
East Asia	2,438,332.3 (2,383,192.5–2,485,969.6)	215.7 (210.8–219.9)	678,297.9 (665,407.7–691,438)	145.7 (142.9–148.5)	−1.39 (-1.53 to−1.24)
Eastern Europe	239,774.2 (234,560.9–245,362)	165.6 (162–169.5)	151,511.2 (148,682.3–154,019.8)	192.8 (189.2–196)	0.54 (0.5 to 0.57)
Eastern Sub-Saharan Africa	1,633,523.7 (1,599,429.5–1,674,402.8)	385.2 (377.1–394.8)	2,409,789.9 (2,354,788.7–2,462,047.5)	348.8 (340.8–356.4)	−0.32 (-0.35 to−0.29)
High-income Asia Pacific	152,565.9 (146,858.2–157,621.3)	161.7 (155.7–167.1)	90,800.9 (88,213.7–93,226.3)	176.2 (171.2–180.9)	0.32 (0.26 to 0.38)
High-income North America	570,389.2 (557,410.5–580,961.1)	259.4 (253.5–264.2)	529,839.5 (521,219.9–538,996.8)	274.9 (270.4–279.7)	0.02 (-0.11 to 0.15)
North Africa and Middle East	1,812,748.6 (1,772,151.6–1,857,533.9)	339.3 (331.7–347.7)	1,906,609.1 (1,859,159–1,955,028.3)	337 (328.7–345.6)	−0.06 (-0.11 to−0.01)
Oceania	39,738.8 (37,276–42,377.6)	353.8 (331.8–377.3)	78,899.9 (73,503.2–84,948.4)	360.7 (336–388.4)	0.07 (0.04 to 0.1)
South Asia	10,098,437.2 (9,951,628–10,250,517.9)	645.6 (636.2–655.3)	7,968,446.2 (7,847,251.4–8,097,713)	530.5 (522.4–539.1)	−0.71 (-0.77 to−0.65)
Southeast Asia	1,710,808.9 (1,679,207.5–1,745,582.4)	287.8 (282.5–293.7)	1,336,968.5 (1,312,458–1,361,660.8)	259.7 (254.9–264.5)	−0.29 (-0.32 to−0.26)
Southern Latin America	101,443 (94,844–108,611.2)	199.3 (186.4–213.4)	84,021 (78,957.1–89,251.5)	244.9 (230.1–260.1)	0.74 (0.7 to 0.78)
Southern Sub-Saharan Africa	247,889.6 (240,080.8–255,590.6)	316.3 (306.3–326.1)	275,889.9 (268,198.4–284,135)	334.2 (324.9–344.2)	0.13 (0.05 to 0.22)
Tropical Latin America	435,227.1 (424,982.2–446,243.5)	261.3 (255.2–267.9)	422,385 (414,428.2–430,792.7)	286.7 (281.3–292.4)	0.37 (0.27 to 0.48)
Western Europe	487,268.9 (473,570.1–502,320.1)	218.7 (212.5–225.4)	465,757.1 (452,408.1–480,445.1)	240.1 (233.2–247.6)	0.32 (0.3 to 0.34)
Western Sub-Saharan Africa	1,500,634.4 (1,478,849.1–1,520,998.4)	351.8 (346.7–356.5)	3,058,057 (3,012,409.8–3,102,868.2)	346 (340.8–351.1)	−0.08 (-0.11 to−0.05)
Deaths					
Global	1,416,179.4 (1,230,955.5–1,661,824.4)	22.5 (19.5–26.4)	623,302.6 (509,686.7–735,006.3)	10.2 (8.4–12)	−2.39 (-2.5 to−2.28)
High SDI	175,487.4 (154,399.6–196,490.4)	11 (9.7–12.3)	27,479.8 (25,209.8–28,923.4)	2.6 (2.4–2.8)	−4.53 (-4.61 to−4.46)
High-middle SDI	225,665.2 (197,448.3–256,462.8)	18.2 (15.9–20.7)	58,770.6 (51,586–66,463.4)	7.1 (6.2–8)	−2.8 (-2.84 to−2.75)
Middle SDI	158,452.1 (136,093.7–182,978.8)	19.4 (16.7–22.4)	61,270.2 (51,095.6–70,849.1)	7.9 (6.6–9.2)	−2.91 (-3 to−2.81)
Low-middle SDI	362,526.6 (288,782.7–442,432.3)	33.7 (26.8–41.1)	152,897.8 (123,084.1–187,628.4)	14.6 (11.8–18)	−2.15 (-2.37 to−1.94)
Low SDI	493,320.8 (396,507.5–622,914.1)	31.4 (25.2–39.6)	322,526.1 (252,757–398,600.7)	13.4 (10.5–16.6)	−2.46 (-2.63 to−2.29)
Andean Latin America	9,858.6 (7,903.5–11,944.9)	17.4 (13.9–21.1)	3,620.7 (3,185.1–4,094.1)	6.6 (5.8–7.5)	−3.51 (-3.97 to−3.06)
Australasia	721 (685.8–788.5)	4.7 (4.5–5.1)	317.1 (285.9–348.8)	1.8 (1.7–2)	−2.03 (-2.47 to−1.59)
Caribbean	6,220.5 (5,438.5–7,138.6)	14.5 (12.7–16.6)	3,209.9 (2,637.4–4,028.4)	8.4 (6.9–10.6)	−1.65 (-1.8 to−1.5)
Central Asia	9,878.8 (8,533.5–11,036.2)	10.3 (8.9–11.6)	9,311.8 (8,578.5–10,062.8)	9.3 (8.6–10.1)	−0.11 (-0.43 to 0.2)
Central Europe	8,781.1 (8,246.2–9,501.2)	10.5 (9.9–11.4)	1,139.4 (1,008.1–1,229.6)	2.4 (2.2–2.6)	−4.32 (-4.44 to−4.2)
Central Latin America	37,745.6 (36,356.3–39,141.2)	15.9 (15.3–16.5)	10,990.7 (10,229.5–11,829.5)	6.1 (5.7–6.6)	−2.83 (-2.99 to−2.66)
Central Sub-Saharan Africa	23,404.9 (15,446.1–31,223)	18.9 (12.4–25.2)	27,620.7 (18,724.9–38,022.2)	11.7 (8–16.2)	−1.3 (-1.42 to−1.18)
East Asia	158,291.2 (116,809.6–208,784.4)	14 (10.3–18.5)	6,223.9 (5,018.3–7,522)	1.3 (1.1–1.6)	−7.52 (-8.11 to−6.93)
Eastern Europe	9,258.7 (8,364.4–10,140.3)	6.4 (5.7–7)	1,413.1 (1,173.5–1,568.8)	1.8 (1.5–2)	−4.07 (-4.34 to−3.8)
Eastern Sub-Saharan Africa	85,731.3 (66,165.8–118,455.6)	20.4 (15.7–28.3)	75,165.1 (58,867.3–92,700)	10.9 (8.5–13.4)	−1.87 (-1.99 to−1.76)
High-income Asia Pacific	3,615.3 (3,292.5–4,137.6)	3.8 (3.5–4.4)	351.5 (289.2–398.3)	0.7 (0.6–0.8)	−5.12 (-5.29 to−4.95)
High-income North America	15,216.7 (14,801.9–15,984.2)	6.9 (6.7–7.3)	6,632.9 (6,178.1–7,091.3)	3.4 (3.2–3.7)	−1.85 (-1.98 to−1.72)
North Africa and Middle East	164,697.3 (140,910.2–191,158.5)	30.9 (26.5–35.9)	52,564.9 (44,532.6–61,928.9)	9.3 (7.9–10.9)	−3.69 (-3.74 to−3.63)
Oceania	1,158.2 (854.8–1,464.4)	10.3 (7.6–13.1)	1,698.5 (1,087.4–2,450.6)	7.8 (5–11.2)	−0.7 (-0.83 to−0.57)
South Asia	631,965.9 (514,219.7–755,235.3)	40.6 (33–48.5)	234,342.1 (196,761.1–277,577)	15.6 (13.1–18.5)	−2.48 (-2.75 to−2.22)
Southeast Asia	98,519.8 (75,463.1–124,592.4)	16.6 (12.7–21)	39,782.6 (29,907.9–49,179.5)	7.7 (5.8–9.5)	−2.52 (-2.59 to−2.44)
Southern Latin America	8,005.4 (7,513.5–8,569.8)	15.7 (14.8–16.8)	1,735.6 (1,603.1–1,914.7)	5 (4.7–5.6)	−3.41 (-3.52 to−3.29)
Southern Sub-Saharan Africa	10,422.5 (8,336.3–12,484.8)	13.3 (10.7–16)	8,474.9 (6,961.2–10,153.3)	10.3 (8.4–12.3)	−0.56 (-0.86 to−0.27)
Tropical Latin America	33,797.6 (32,130.7–35,465.9)	20.3 (19.3–21.3)	6,002.5 (5,627–6,366.2)	4.1 (3.8–4.3)	−4.76 (-4.93 to−4.6)
Western Europe	10,624.1 (10,166.9–11,616)	4.8 (4.5–5.2)	3,675.9 (3,270.9–3,906.3)	1.9 (1.7–2)	−2.64 (-2.89 to−2.38)
Western Sub-Saharan Africa	88,265.3 (62,306.6–121,950.9)	21 (14.8–29.1)	129,028.6 (94,731.5–173,887.2)	14.7 (10.8–19.8)	−1.03 (-1.13 to−0.94)
Disability-adjusted life years					
Global	135,303,930.7 (117,894,781.4–156,892,962.7)	2,160.4 (1,881.6–2,502.9)	70,218,511 (60,636,450.8–81,370,219.9)	1,101 (947.2–1,281.4)	−2.03 (-2.14 to−1.92)
High SDI	17,931,803.7 (15,959,342–20,007,726.9)	1,097 (973.5–1,225)	4,899,273.8 (4,290,524.6–5,600,579.1)	344.7 (313.1–380.7)	−3.68 (-3.75 to−3.61)
High-middle SDI	21,757,323 (19,075,302.8–24,719,696)	1,753.7 (1,537.6–1,991.9)	8,132,797.5 (7,270,282.7–9,267,838.7)	821.6 (732.7–921.8)	−2.22 (-2.27 to−2.17)
Middle SDI	15,074,491.5 (12,913,572.1–17,218,408)	1,869 (1,603.7–2,129)	6,878,889 (5,993,457.2–7,812,992.1)	855.5 (741.6–969.6)	−2.53 (-2.62 to−2.44)
Low-middle SDI	34,314,892.1 (27,520,197.8–41,555,742.4)	3,234.2 (2,610–3,908.9)	17,427,293.7 (14,694,124.5–20,313,627)	1,616.3 (1,359.1–1,886.4)	−1.77 (-1.97 to−1.56)
Low SDI	46,153,002.6 (37,384,686.3–57,768,477.4)	3,007.7 (2,446.5–3,749.5)	32,840,194.5 (26,595,288.3–39,849,011)	1,409.6 (1,156.5–1,711.1)	−2.18 (-2.35 to−2.01)
Andean Latin America	942,349.3 (768,652.8–1,130,307.4)	1,694.6 (1,391.5–2,027.1)	394,528.2 (348,830–444,609.9)	697.7 (616.2–785.7)	−3.17 (-3.58 to−2.76)
Australasia	93,995.5 (84,315.4–104,669.5)	569.2 (516.4–626.2)	65,469.4 (55,036.8–77,800.4)	291 (251.4–336.7)	−1.48 (-1.79 to−1.17)
Caribbean	633,677.7 (558,938.5–716,523)	1,499.5 (1,322.3–1,694.1)	364,350 (307,588.4–435,965.3)	921.6 (777.2–1,110.8)	−1.46 (-1.59 to−1.33)
Central Asia	958,931 (831,661.4–1,068,545)	1,022.9 (887.5–1,136.9)	921,468.5 (854,718.5–1,000,178.7)	921.8 (854.9–1,000.4)	−0.12 (-0.41 to 0.17)
Central Europe	922,885.9 (860,297.6–992,647.1)	1,054.3 (988.8–1,136.7)	209,815.7 (180,672.9–239,003.7)	326.3 (288.2–362.2)	−3.49 (-3.64 to−3.35)
Central Latin America	3,573,736.1 (3,411,542.6–3,721,209.2)	1,526 (1,454.2–1,592.1)	1,266,208 (1,158,344.9–1,371,967.1)	659.5 (607.9–709.2)	−2.47 (-2.63 to−2.32)
Central Sub-Saharan Africa	2,146,012 (1,427,139.9–2,849,970.7)	1,752.3 (1,171–2,318.6)	2,623,255.5 (1,854,190.2–3,538,751.6)	1,134.1 (806.1–1,520.4)	−1.17 (-1.27 to−1.06)
East Asia	15,119,192.8 (11,444,266.1–19,565,523.4)	1,329.1 (1,001.9–1,722.7)	1,429,634.3 (1,173,474.2–1,688,377.5)	182.5 (154–214.6)	−6.45 (-6.87 to−6.03)
Eastern Europe	982,732.5 (892,532.8–1,068,953.1)	642.4 (583.2–695.4)	254,792.3 (218,285.7–293,389.6)	233.8 (201.3–262.6)	−3.32 (-3.52 to−3.12)
Eastern Sub-Saharan Africa	7,886,790.1 (6,109,157.3–10,842,298.8)	1,910.5 (1,486.3–2,620.2)	7,412,465.9 (5,962,408.9–9,076,892.8)	1,103.1 (891.5–1,350.3)	−1.6 (-1.73 to−1.47)
High-income Asia Pacific	457,685.2 (406,168.6–521,568.9)	422.8 (380.1–478.5)	158,520.6 (124,133.2–193,197.8)	142.3 (118.1–168.1)	−3.23 (-3.41 to−3.05)
High-income North America	1,787,942 (1,672,041.7–1,920,821.6)	777.8 (731.9–829.2)	1,092,850.5 (973,602.1–1,239,299.7)	459.3 (416.5–507.9)	−1.4 (-1.5 to−1.3)
North Africa and Middle East	15,451,200.6 (13,238,734.5–17,885,420)	2,950.5 (2,532.6–3,412.7)	5,701,209.4 (4,991,615.3–6,535,493.1)	982.6 (857.8–1,129.2)	−3.36 (-3.42 to−3.31)
Oceania	115,505.9 (88,363.9–145,337.4)	1,078.2 (835.5–1,354.8)	174,199.4 (120,116–243,664.1)	830.7 (582–1,153)	−0.64 (-0.75 to−0.53)
South Asia	60,405,287.3 (49,504,978.8–71,541,140.5)	3,934.6 (3,233.2–4,650.6)	28,839,423.6 (24,858,464.1–33,528,635.5)	1,805 (1,550.5–2,100.5)	−2 (-2.24 to−1.76)
Southeast Asia	9,454,529.3 (7,432,987.7–11,799,081.5)	1,608.3 (1,267.1–2,004.1)	4,423,483.5 (3,502,027.8–5,361,944)	815 (640.5–992.5)	−2.22 (-2.29 to−2.15)
Southern Latin America	780,960.4 (729,626.5–835,869.3)	1,535.4 (1,434.4–1,643.2)	241,181.3 (218,135–271,500.5)	582.1 (532.3–639.4)	−2.89 (-3.02 to−2.76)
Southern Sub-Saharan Africa	996,455.9 (805,921.2–1,188,720.5)	1,293.2 (1,048.6–1,538.5)	873,497.6 (737,601.1–1,029,244)	1,044.6 (881.2–1,231)	−0.41 (-0.67 to−0.15)
Tropical Latin America	3,178,102.9 (3,019,359.5–3,327,597.6)	1,909.5 (1,814.2–1,999.3)	850,559.1 (761,623–943,914.7)	515.6 (472.3–564.2)	−3.95 (-4.11 to−3.8)
Western Europe	1,333,518.2 (1,215,325–1,451,489.7)	532.6 (494.3–575.6)	745,648.7 (637,016–857,775.7)	275.9 (244–309.8)	−1.83 (-2.04 to−1.62)
Western Sub-Saharan Africa	8,082,439.9 (5,747,245–11,131,769.9)	1,947.7 (1,395.9–2,687.8)	12,175,949.6 (9,041,747.9–16,270,939.6)	1,413.6 (1,053.8–1,883.7)	−0.91 (-1.01 to−0.81)

### Regional level

The global burden of preterm birth demonstrates marked regional disparities, closely associated with the SDI levels. ASPR highlight significant differences across regions. In 2023, High SDI regions reported the lowest ASPR at 1,120.3 per 100,000 population (95% UI: 986.6–1,267.3), whereas Low-middle SDI regions exhibited the highest rate at 2,433.4 per 100,000 (95% UI: 2,109.7–2,804.5) (Table 1). Notably, High-middle SDI regions experienced the most significant increase in burden over time, with an EAPC of 0.97 (95% CI: 0.92 to 1.02), a trend possibly driven by factors such as rising maternal age and an increase in medically indicated preterm births associated with more complex pregnancies. In these regions, the ASPR rose from 1,212.2 per 100,000 (95% UI: 1,038.0–1,380.3.3) in 1990 to 1,592.9 per 100,000 (95% UI: 1,398.1–1,790.2) in 2023, indicating a growing public health challenge. Conversely, Low SDI regions showed the most substantial decline in ASPR, with an EAPC of −0.27 (95% CI: −0.3 to −0.24). This decline suggests potential progress in prevention and management, although it must be interpreted with caution, as it may also be influenced by under-reporting or variations in data quality over the 33-year period. The rate decreased from 2,084.6 per 100,000 (95% UI: 1,716.5–2,482.6) in 1990 to 1,924.7 per 100,000 (95% UI: 1,643.5–2,236.3) in 2023, suggesting some progress in the prevention and management of preterm births. These trends likely reflect a complex interplay of factors, including improved surveillance and reporting systems, rising maternal age and associated obstetric complications, and expanded access to healthcare services, which may enhance the detection of preterm births.

Regional variations in the ASIR and ASDR further highlight the persistent disparities in preterm birth outcomes across different SDI levels. Low-middle SDI regions reported the highest ASIR of preterm birth, whereas High-middle SDI regions had the lowest. Specifically, the ASIR in Low-middle SDI regions was 427.6 per 100,000 population (95% UI: 416.5–438.2), compared to 231.0 per 100,000 (95% UI: 228.4–233.5) in High SDI regions. The disparity was even more pronounced in ASDR. In 2023, the ASDR in Low-SDI regions (13.4 per 100,000) was over five times higher than in High-SDI regions (2.6 per 100,000), revealing a profound survival gap. The burden of disease, as measured by the age-standardized DALY rate, further underscores these regional disparities. Low-middle SDI regions bore the highest burden, with a DALY rate of 1,616.3 per 100,000 (95% UI: 1,359.1–1,886.4), while High SDI regions reported the lowest rate at 344.7 per 100,000 (95% UI: 313.1–380.7) (Table 1). These findings collectively highlight the pronounced inequities in preterm birth outcomes across SDI levels, reflecting the complex interplay between socio-demographic development and health outcomes.

The burden of preterm birth was most concentrated in the South Asia region, which not only had the highest ASPR at 3,125.0 per 100,000 population (95% UI: 2,694.5–3,620.7) but also accounted for a disproportionate more than 10% of global DALYs in 2023 (Table 1). A disproportionately high burden was also evident in several smaller regions. The Caribbean and Oceania, for instance, reported high ASPRs of 1,763.2 (95% UI: 1,468.7–2,201.5) and 1,651.1 (95% UI: 1,275.2–2,359.7) per 100,000, respectively. These rates are likely driven by a combination of limited healthcare access and persistent socioeconomic and clinical risk factors common to these areas. In terms of incidence, South Asia and the Caribbean were also among the regions with the highest ASIRs of preterm birth globally. South Asia reported an ASIR of 530.5 per 100,000 (95% UI: 522.4–539.1), while the Caribbean followed with 388.2 per 100,000 (95% UI: 373.1–404.6). In contrast, East Asia recorded the lowest ASIR at 145.7 per 100,000 (95% UI: 142.9–148.5). Notably, temporal trends from 1990 to 2023 revealed that East Asia experienced the most substantial decline in ASIR, with an EAPC of −1.39 (95% CI: −1.53 to −1.24). Conversely, Southern Latin America exhibited the most significant increase in ASIR, with an EAPC of 0.74 (95% CI: 0.70 to 0.78) (Table 1, Figure 1). This rise is likely associated with key demographic shifts in the region, primarily the trend toward delayed childbearing and a corresponding increase in high-risk pregnancies. These contrasting trends underscore the complex and regionally variable dynamics of preterm birth incidence, highlighting the necessity for context-specific public health strategies and continued research to address these disparities. ASDRs for preterm birth were also disproportionately high in several regions. South Asia, Western Sub-Saharan Africa and Central Sub-Saharan Africa all reported ASDRs exceeding 11 per 100,000 population. From 1990 to 2023, the most substantial reduction in ASDR was observed in East Asia, with an EAPC of −7.52 (95% CI: −8.11 to −6.93), this remarkable success is likely attributable to a combination of factors, including rapid expansion of universal health coverage, significant investments in maternal health programs, and the widespread establishment of advanced NICUs in the region over the past three decades (Table 1). Despite overall global declines, the burden of disease, as measured by the age-standardized DALY rate, remained disproportionately concentrated in South Asia and Western Sub-Saharan Africa. In 2023, South Asia recorded the highest rate at 1,805.0 per 100,000 (95% UI: 1,550.5–2,100.5), followed closely by Western Sub-Saharan Africa at 1,413.6 per 100,000 (95% UI: 1,053.8–1,883.7) (Table 1), highlighting these regions as persistent epicenters of the preterm birth burden. Between 1990 and 2023, the most significant reductions in DALY rates were seen in East Asia (EAPC: −6.45, 95% CI: −6.87 to −6.03) and Tropical Latin America (EAPC: −3.95, 95% CI: −4.11 to −3.80) (Table 1). This success is likely attributable to the effective implementation and scaling-up of targeted interventions, including widespread improvements in both maternal and neonatal care.

### National level

**Figure 2:**
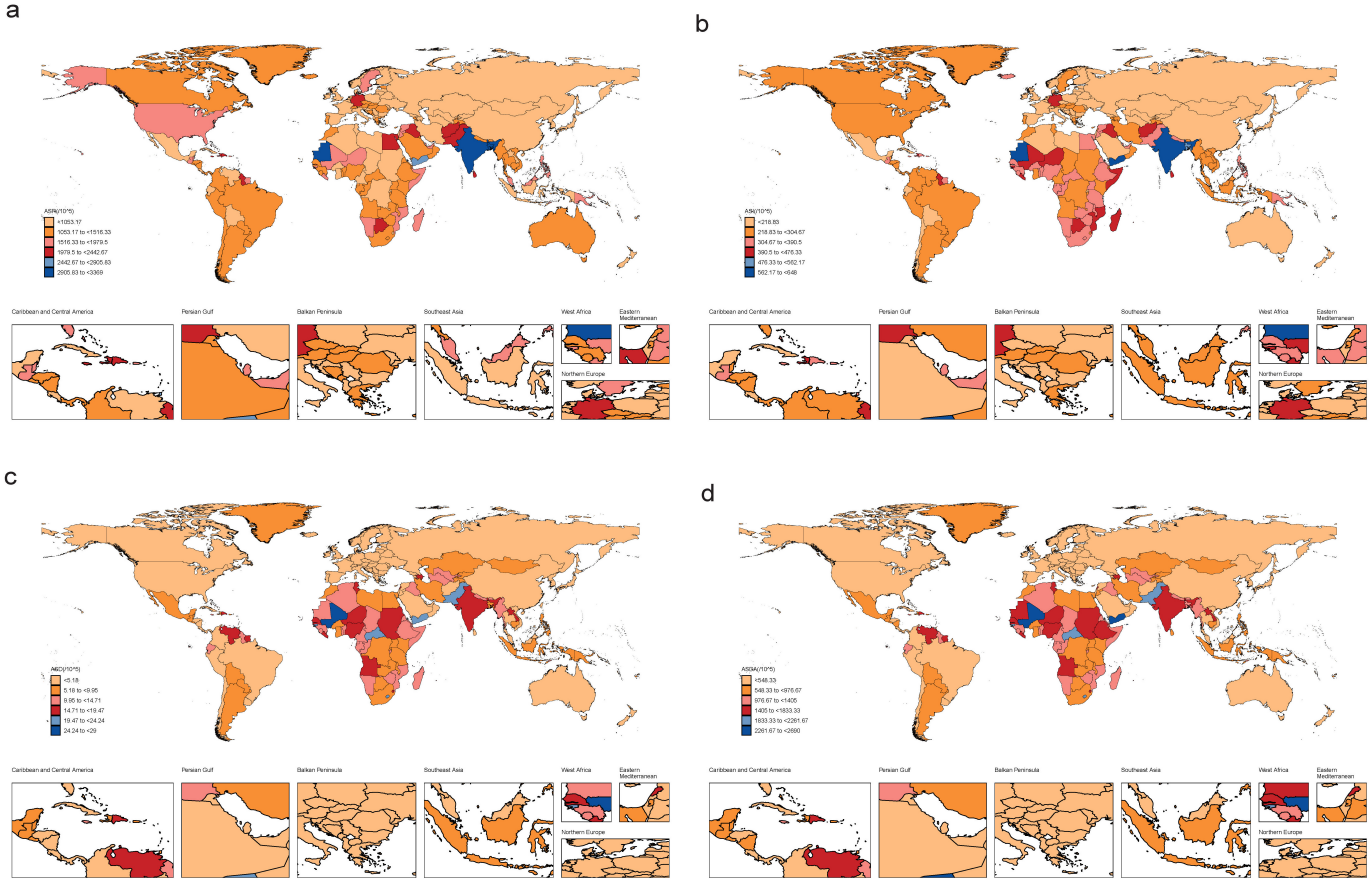
The global disease burden of preterm birth for both sexes in 204 countries and territories. **(a)** Prevalence rate, **(b)** Incidence rate, **(c)** Death rate, **(d)** DALYs rate.

The ASPR of preterm birth in 2023 exhibited considerable global variation, ranging from approximately 596 to 3,368 per 100,000 individuals. Among all countries, the highest ASPRs were observed in India (3,368.4 per 100,000; 95% UI: 2,902.0–3,892.8), Mauritania (3,358.3 per 100,000; 95% UI: 2,687.9–4,336.1), and Bangladesh (3,107.5 per 100,000; 95% UI: 2,539.4–3,858.6) (Figure 2a, Supplementary Table S1). The disproportionate impact of these countries is further underscored by the fact that these three nations alone collectively accounted for approximately 3.6% of the total global DALYs due to preterm birth in 2023. These findings are consistent with regional analyses, which identified South Asia as the region bearing the highest global burden of preterm birth prevalence. The country-specific distribution of the ASIR is presented in Figure 2b and Supplementary Table S2. Mauritania recorded the highest ASIR at 647.9 per 100,000 individuals (95% UI: 610.5–693.9), while Tonga reported the lowest ASIR at 134.3 per 100,000 (95% UI: 119.1–149.1). Analysis of the ASDR and DALYs due to preterm birth further highlights the countries most severely affected. Mali ranked highest in both ASDR (28.8 per 100,000; 95% UI: 22.5–35.2) and DALY rate (2,689.2 per 100,000; 95% UI: 2,117.3–3,291.5). Yemen followed, with the second highest ASDR (23.4 per 100,000; 95% UI: 17.7–29.6) and DALY rate (2,289.0 per 100,000; 95% UI: 1,749.4–2,847.5) (Figures 2c, d, Supplementary Tables S3, S4). This distinction is not surprising, as it directly reflects the devastating impact of intersecting crises in these nations, including collapsed or severely weakened neonatal care systems, the effects of prolonged armed conflict, and extreme poverty, all of which create a perfect storm for preventable neonatal deaths. The considerable overlap among countries with the highest ASDR and DALY rates suggests a disproportionately high burden of preterm birth in these regions, significantly impacting both mortality and health-related quality of life.

**Figure 3:**
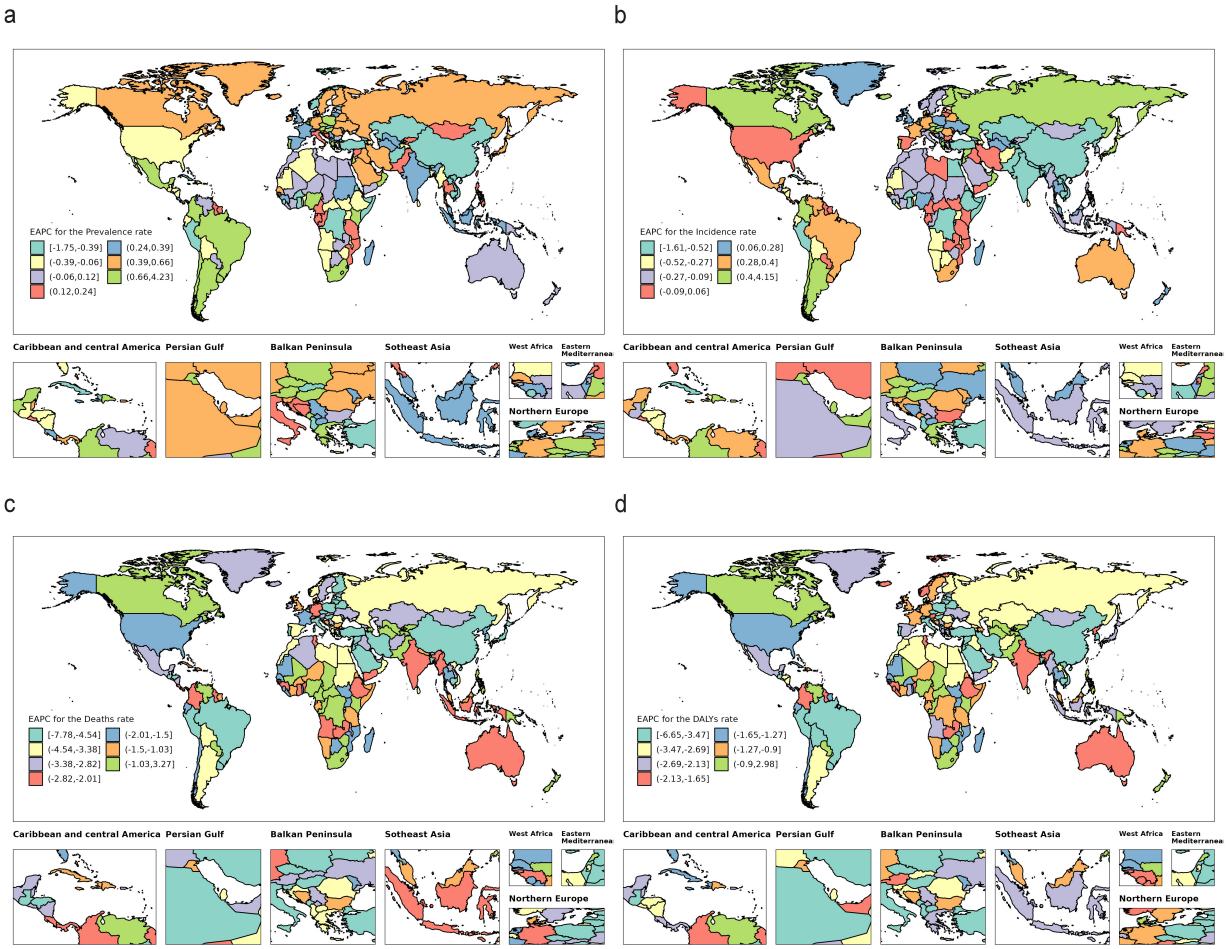
Change cases of preterm birth for both sexes in 204 countries and territories. **(a)** Change prevalence cases. **(b)** Change incidence cases. **(c)** Change deaths cases. **(d)** Change DALYs.

The Greece exhibited the most pronounced increase in prevalence cases, with a growth rate of 4.23 (Figure 3a, Supplementary Table S5), possibly due to demographic transitions like delayed childbearing, whereas the Burkina Faso demonstrated the largest reduction, with a decrease of−1.75, likely reflecting improvements in primary maternal healthcare. Similarly, in terms of incidence cases, the Greece again recorded the highest increase (4.15), while the Federal Democratic Republic of Nepal experienced the greatest decline (-1.61) (Figure 3b, Supplementary Table S6). Regarding mortality, the Republic of Uzbekistan showed the most substantial rise in the number of death cases (3.27), in contrast to China, which reported the largest decrease (-7.78) (Figure 3c, Supplementary Table S7), a success likely driven by its massive expansion of neonatal intensive care and universal health coverage. Finally, the Republic of Uzbekistan also registered the highest increase in DALYs, with a rise of 2.98, while China exhibited the most significant reduction, at −6.65 (Figure 3d, Supplementary Table S8).

### Age and sex patterns

**Figure 4:**
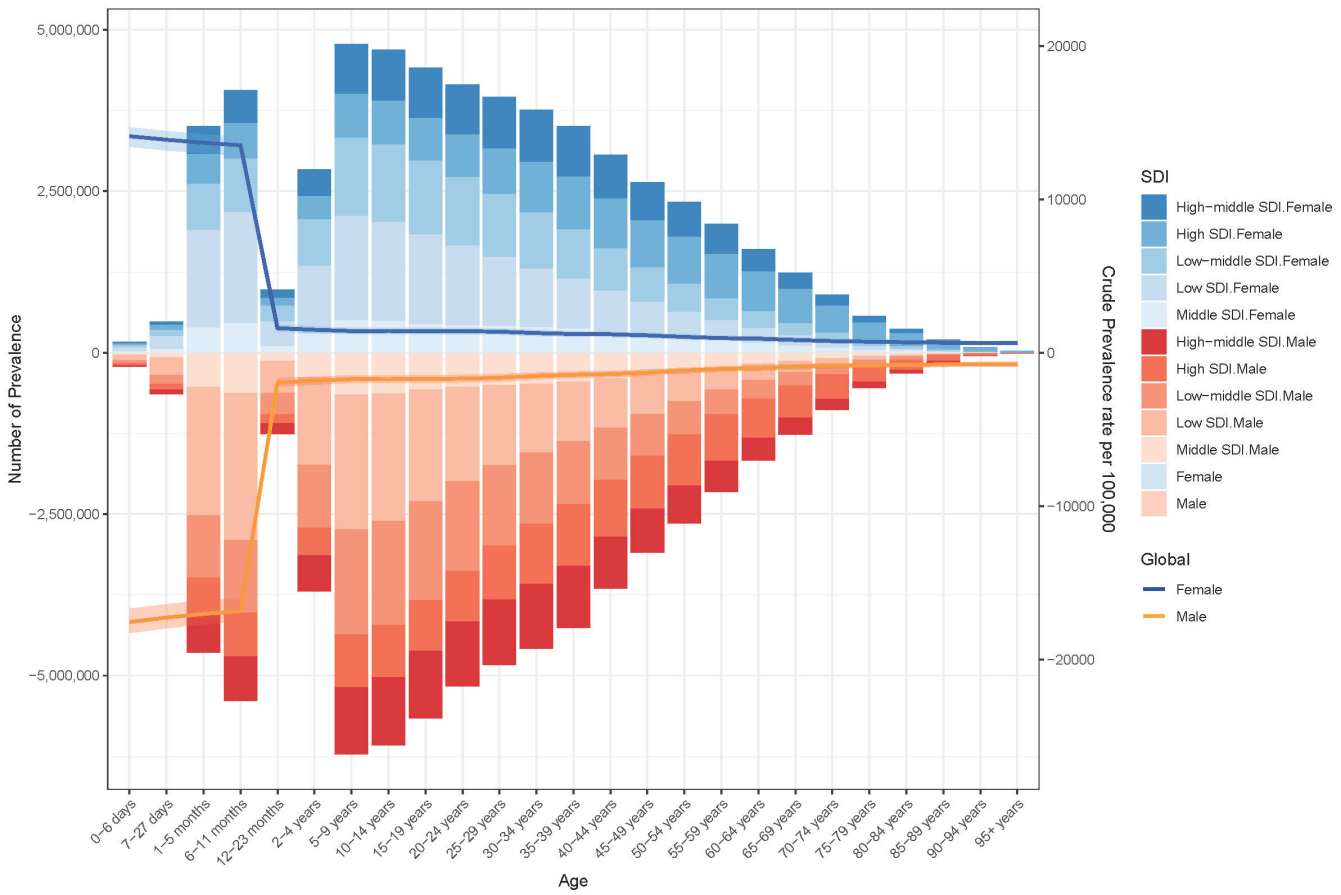
The age-specific numbers and ASPRs of preterm birth by SDI regions in 2023.

**Figure 5:**
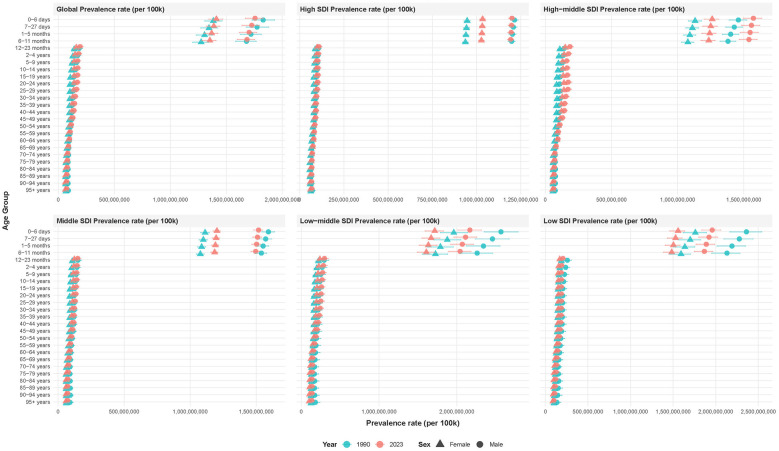
Age-standardized prevalence rates of preterm birth by sex, age group, and socio-demographic index, 1990 and 2023.

In 2023, the ASPR of preterm birth was highest in infants under 11 months, with a secondary peak in the 5–9 year age group, and then declined progressively with increasing age after the 10–14 year age group (Figure 4). The ASPR values were comparable between males and females, indicating no substantial sex-based differences in prevalence, a finding inconsistent with previous GBD analyses (15). Both the ASDR and DALYs associated with preterm birth declined in 2023 for both sexes. However, these metrics remained highest in the 0–6 days and 7–27 days age group, underscoring the neonatal period (< 28 days) as the critical window for interventions aimed at reducing death and disability. (Supplementary Figures S1, S2). A crucial finding emerged from the SDI-stratified age analysis. While the prevalence of infants under 1 year was high across all SDI regions, a stark survival gap was evident (Figure 5). The age-standardized death rate for neonates (< 28 days) in Low-SDI regions was substantially higher than in High-SDI regions, demonstrating that while preterm birth is a global problem, survival is largely determined by access to advanced neonatal care available in wealthier settings (Supplementary Figures S3–S4).

### Future projections of the global burden of preterm birth

**Figure 6:**
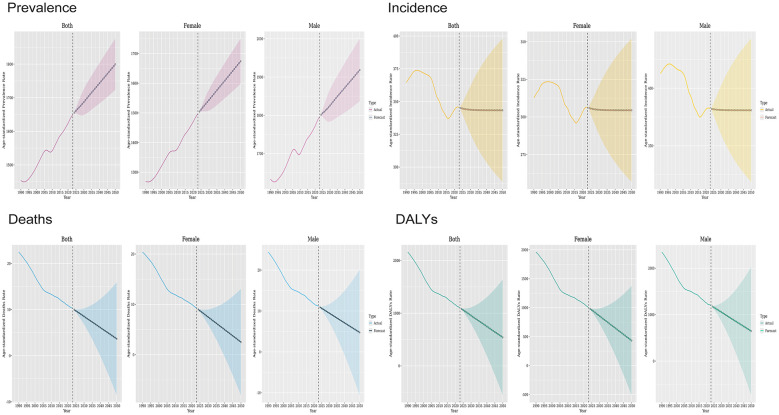
Future forecasts of global burden of preterm birth.

The global burden of preterm birth is projected to undergo notable changes by 2050, with varying trends across key epidemiological indicators. According to current forecasts, the ASPR for preterm birth is expected to increase globally for both sexes combined, rising from 1,649.5 per 100,000 population in 2023 to 1,799.4 per 100,000 by 2050. This represents an approximate 9.1% increase, driven primarily by anticipated demographic shifts, including rising maternal age in many regions (Figure 6). When stratified by sex, the prevalence among males is projected to show a modest increase from 1,796.3 per 100,000 in 2023 to 1,918.6 per 100,000 in 2050. In contrast, females are expected to experience a more substantial rise, with ASPR increasing from 1,497.2 to 1,674.4 per 100,000 over the same period, indicating a steeper upward trend in the female population. This difference is likely linked to the established survival advantage of female neonates, which may become more pronounced in population-level prevalence as overall neonatal survival continues to improve. The global ASIR is also anticipated to decline slightly for both sexes combined, from 345.6 per 100,000 in 2023 to 343.3 per 100,000 by 2050, suggesting a relatively stable trend in the occurrence of preterm birth. Conversely, the ASDR is projected to decline markedly, decreasing from 10.2 per 100,000 in 2023 to 3.7 per 100,000 by 2050. This decline may reflect anticipated improvements in neonatal care, early detection, and clinical management of preterm complications. Similarly, the age-standardized DALY rate is forecasted to decrease significantly, from 1,101.0 per 100,000 population in 2023 to 541.2 per 100,000 in 2050. This projected progress hinges on continued advancements in the continuum of neonatal care, from health system strengthening to the deployment of life-saving technologies. These projections highlight a complex global pattern: while the prevalence and incidence of preterm birth may continue to rise modestly, particularly among females, the associated mortality and DALY burden are expected to decline significantly, reflecting improved survival but persistent risk of preterm delivery.

## Discussion

This study provides a comprehensive analysis of the global, regional, and national burden of preterm birth from 1990 to 2023 using estimates derived from the GBD 2023 framework. Despite notable declines in age-standardized incidence, mortality, and DALY rates, the absolute global burden of preterm birth has increased substantially over the past three decades. These trends reflect the complex interplay between demographic shifts, health system performance and sociodemographic development.

Globally, the number of prevalent cases of preterm birth increased by 54.0% from 1990 to 2023, reaching over 125 million cases in 2023. This increase was largely driven by population growth and improved survival of preterm infants. Although the ASPR showed only a modest rise (EAPC 0.40), the ASIR declined slightly (EAPC −0.33), indicating some progress in primary prevention, antenatal care, or reporting practices. These findings are consistent with prior GBD studies that have shown population aging and improved neonatal survival contributing to a higher absolute burden of neonatal conditions, despite relative improvements in standardized rates (16, 17). Encouragingly, both the ASDR and age-standardized DALY rate declined significantly over the study period (EAPCs −2.39 and −2.03, respectively), reflecting advances in neonatal intensive care, early diagnosis, and maternal health services across many parts of the world. Nevertheless, the persistence of high absolute mortality (623,303 deaths in 2023) and DALYs (70.2 million) highlights that preterm birth remains a leading cause of neonatal death and lifelong disability.

A striking feature of our findings is the marked disparity in preterm birth burden across SDI levels. Low and low-middle SDI regions consistently exhibited higher ASPR, ASIR, ASDR, and DALY rates compared to high SDI regions. For example, the ASDR in low SDI regions was nearly five times that of high SDI regions (13.4 vs. 2.6 per 100,000), and the DALY rate in low-middle SDI regions was more than four times higher than in high SDI regions. These disparities underscore critical weaknesses in health system capacity in low-SDI settings. They point to significant policy gaps related to ensuring universal access to a continuum of care, from preventative maternal health services to advanced neonatal intensive care, which remains a distant reality in many high-burden regions (1, 18).

Regionally, India, Mauritania, and Bangladesh had the highest prevalence rates, while Mauritania also had the highest incidence. These areas are characterized by a combination of health system limitations, socioeconomic inequality, high rates of adolescent pregnancy, and elevated exposure to maternal risk factors such as malnutrition, infections, and air pollution (19–21). The countries with the highest mortality and DALY rates: Mali, Yemen, and Lesotho, also had the weakest health systems and highest maternal and neonatal mortality rates globally, underscoring the urgent need for targeted policy interventions in fragile health systems. The burden of preterm birth was highest during the neonatal period (< 28 days), as expected given the direct link between preterm birth and neonatal death. While ASPRs were relatively stable across sexes, ASDR and DALY rates were higher among neonates and decreased with age, reflecting the critical vulnerability of this group.

The projected trends in the global burden from 2023 to 2050 highlight both progress and persisting challenges in neonatal health. The anticipated increase in the ASPR, particularly among females, suggests that while survival rates may be improving, the overall number of preterm births remains a growing concern. This is likely a direct consequence of the known survival advantage of female neonates; as overall survival from preterm birth improves, the greater resilience of females leads to a disproportionately faster increase in their prevalence within the surviving population. This rise could also be influenced by broader demographic shifts, such as rising maternal age. (1, 22). Notably, the relatively stable ASIR indicates that while the occurrence of new cases is not rising sharply, the burden remains significant. Encouragingly, the projected decline in the ASDR reflects advancements in neonatal care and early intervention strategies, which may be contributing to improved survival outcomes for preterm infants. Similarly, the substantial reduction in the age-standardized DALY rate suggests a decreasing impact of preterm birth on long-term health and disability, potentially due to better access to healthcare and enhanced management of neonatal complications. However, the overall increasing prevalence underscores the need for continued efforts in prevention strategies, including addressing maternal health, reducing environmental exposures, and improving access to quality antenatal care, particularly in low- and middle-income countries where the burden is often highest (9).

The findings of this study carry significant implications for public health policy, directly informing strategies to meet the Every Newborn Action Plan targets. First, achieving SDGs will require strengthening health systems in low- and middle-income countries by expanding equitable access to high-quality antenatal care, promoting facility-based deliveries, and enhancing neonatal care services. Second, targeted interventions to address modifiable maternal risk factors, such as improving maternal nutrition, reducing the prevalence of adolescent pregnancies, and effectively managing chronic health conditions, should be prioritized within national maternal and child health agendas. Third, countries exhibiting increasing trends in preterm birth should be prioritized for comprehensive health system assessments and rigorous evaluations of existing interventions, to identify context-specific gaps and inform evidence-based policy responses.

### Limitations

This study is strengthened by the use of GBD 2023 data, which allows for comprehensive and comparable burden estimates across countries and over time. The use of EAPC metrics facilitates interpretation of long-term trends in age-standardized rates. However, several limitations should be noted. First, data quality and completeness vary substantially across countries, particularly in low-income settings where vital registration and health surveillance systems may be weak. Second, the estimates are fundamentally dependent on GBD's complex modeling assumptions. This means that for data-sparse regions, the results are heavily influenced by the model's predictions based on covariates, and while 95% uncertainty intervals are provided to quantify this, the potential for model-based error remains. Third, the attribution of burden to specific risk factors relies on available evidence and may not capture the full complexity of causal pathways, especially in multi-factorial conditions like preterm birth.

## Conclusion

Despite modest improvements in age-standardized incidence, mortality, and DALY rates, the global burden of preterm birth remains substantial and continues to rise in absolute terms. Stark regional and national disparities persist, with South Asia and Sub-Saharan Africa facing the highest burden. These findings underscore the need for intensified efforts to reduce the incidence and impact of preterm birth, particularly in low- and middle-SDI regions. Tailored interventions addressing maternal health and neonatal care infrastructure must be prioritized. Continued global monitoring, investment in data systems, and research will be essential to achieving improvements in neonatal health outcomes. This includes not only research into more effective prevention strategies but also fundamental research into the mechanisms of preterm birth and clinical research to improve treatments and long-term outcomes for survivors.

## Data Availability

The original contributions presented in the study are included in the article/Supplementary material, further inquiries can be directed to the corresponding author.

## Supplementary material

Supplementary Figure 1 The age-specific numbers and ASDRs of preterm birth by SDI regions in 2023.

Supplementary Figure 2 The age-specific numbers and DALYs of preterm birth by SDI regions in 2023.

Supplementary Figure 3 Age-standardized deaths rates of preterm birth by sex, age group, and socio-demographic index, 1990 and 2023.

Supplementary Figure 4 Age-standardized DALYs rates of preterm birth by sex, age group, and socio-demographic index, 1990 and 2023.

Supplementary Tables S1–S4 The global disease burden of preterm birth for both sexes in 204 countries and territories. (1) ASPR, (2) ASIR, (c) ASDR, (d) ASDALR.

Supplementary Tables S5–S8 Change cases of preterm birth for both sexes in 204 countries and territories. (5) Change prevalence cases. (6) Change incidence cases. (7) Change deaths cases. (8) Change DALYs.

## References

[1] Chawanpaiboon S Vogel JP Moller AB Lumbiganon P Petzold M Hogan D . Global, regional, and national estimates of levels of preterm birth in 2014: a systematic review and modelling analysis. Lancet Glob Health. (2019) 7:e37–46. doi: 10.1016/S2214-109X(18)30451-0 30389451 PMC6293055

[2] De Costa A Moller AB Blencowe H Johansson EW Hussain-Alkhateeb L Ohuma EO . Study protocol for WHO and UNICEF estimates of global, regional, and national preterm birth rates for 2010 to 2019. PLoS ONE. (2021) 16:e0258751. doi: 10.1371/journal.pone.0258751 34669749 PMC8528299

[3] Lawn JE Ohuma EO Bradley E Idueta LS Hazel E Okwaraji YB . Small babies, big risks: global estimates of prevalence and mortality for vulnerable newborns to accelerate change and improve counting. Lancet. (2023) 401:1707–19. doi: 10.1016/S0140-6736(23)00522-6 37167989

[4] Alliance for M Newborn Health Improvement mortality study group. Population-based rates, timing, and causes of maternal deaths, stillbirths, and neonatal deaths in south Asia and sub-Saharan Africa: a multi-country prospective cohort study. Lancet Glob Health. (2018) 6:e1297-e308. doi: 10.1016/S2214-109X(18)30385-1 30361107 PMC6227247

[5] Sefidkar R Zayeri F Kazemi E Salehi M Dehnad A Hafizi M. Trend study of preterm infant mortality rate in developed and developing countries over 1990 to 2017. Iran J Public Health. (2021) 50:369–75. doi: 10.18502/ijph.v50i2.5353 33748001 PMC7956079

[6] Liang X Lyu Y Li J Li Y Chi C. Global, regional, and national burden of preterm birth, 1990-2021: a systematic analysis from the global burden of disease study 2021. EClinicalMedicine. (2024) 76:102840. doi: 10.1016/j.eclinm.2024.102840 39386159 PMC11462015

[7] Moutquin JM. Classification and heterogeneity of preterm birth. BJOG. (2003) 110:30–3. doi: 10.1046/j.1471-0528.2003.00021.x 12763108

[8] Kadivnik M Kralik K Sijanovic S Wagner J. Genetic and environmental determinants of spontaneous preterm birth: focus on progesterone receptor gene variants. Int J Mol Sci. (2025) 26:10659. doi: 10.3390/ijms262110659 41226694 PMC12609070

[9] Swarray-Deen A Sepenu P Mensah TE Kamara SF Jalloh MB Koroma MM . Preterm birth in low-middle income Countries. Best Pract Res Clin Obstet Gynaecol. (2024) 95:102518. doi: 10.1016/j.bpobgyn.2024.102518 38937155

[10] Ward VC Lee AC Hawken S Kramer MS Shah PS. Overview of the Global and US Burden of Preterm Birth. Clin Perinatol. (2024) 51:301–11. doi: 10.1016/j.clp.2024.02.015 38705642

[11] Vogel JP Chawanpaiboon S Moller AB Watananirun K Bonet M Lumbiganon P. The global epidemiology of preterm birth. Best Pract Res Clin Obstet Gynaecol. (2018) 52:3–12. doi: 10.1016/j.bpobgyn.2018.04.003 29779863

[12] Pravia CI Benny M. Long-term consequences of prematurity. Cleve Clin J Med. (2020) 87:759–67. doi: 10.3949/ccjm.87a.19108 33229393

[13] Rudd KE Johnson SC Agesa KM Shackelford KA Tsoi D Kievlan DR . Global, regional, and national sepsis incidence and mortality, 1990-2017: analysis for the Global Burden of Disease Study. Lancet. (2020) 395:200–11. doi: 10.1016/S0140-6736(19)32989-7 31954465 PMC6970225

[14] Clegg LX Hankey BF Tiwari R Feuer EJ Edwards BK. Estimating average annual per cent change in trend analysis. Stat Med. (2009) 28:3670–82. doi: 10.1002/sim.3733 19856324 PMC2843083

[15] Zhang Z Li L Pan D Liu T Ju X Li Q . Sex differences in preterm birth and the impact of particulate matter pollution: A retrospective cross-sectional study of the Global Burden of Disease 2021. Sci Prog. (2025) 108:368504251387238. doi: 10.1177/00368504251387238 41091820 PMC12536127

[16] Diseases GBD Injuries C. Global burden of 369 diseases and injuries in 204 countries and territories, 1990-2019: a systematic analysis for the Global Burden of Disease Study 2019. Lancet. (2020) 396:1204–22. doi: 10.1016/S0140-6736(20)30925-9 33069326 PMC7567026

[17] Diseases GBD Injuries C. Global incidence, prevalence, years lived with disability (YLDs), disability-adjusted life-years (DALYs), and healthy life expectancy (HALE) for 371 diseases and injuries in 204 countries and territories and 811 subnational locations, 1990-2021: a systematic analysis for the Global Burden of Disease Study 2021. Lancet. (2024) 403:2133–61. doi: 10.1016/S0140-6736(24)00757-8 38642570 PMC11122111

[18] Lawn JE Blencowe H Oza S You D Lee AC Waiswa P . Every Newborn: progress, priorities, and potential beyond survival. Lancet. (2014) 384:189–205. doi: 10.1016/S0140-6736(14)60496-7 24853593

[19] Jejaw M Demissie KA Tafere TZ Tiruneh MG Hagos A Teshale G. Prevalence and Determinants of Early Adolescent Childbearing in High Maternal Mortality Sub-Saharan Africa: A Multilevel Analysis of DHS Data. J Epidemiol Glob Health. (2025) 15:63. doi: 10.1007/s44197-025-00409-7 40261517 PMC12014889

[20] Bezie MM Asebe HA Asnake AA Fente BM Negussie YM Asmare ZA . Factors associated with perinatal mortality in sub-Saharan Africa: A multilevel analysis. PLoS ONE. (2024) 19:e0314096. doi: 10.1371/journal.pone.0314096 39570972 PMC11581209

[21] Mate A Reyes-Goya C Santana-Garrido A Vazquez CM. Lifestyle, Maternal Nutrition and Healthy Pregnancy. Curr Vasc Pharmacol. (2021) 19:132–40. doi: 10.2174/18756212MTA1DNTgkw 32234002

[22] Yu Z Zhang X Zhang J Feng Y Zhang H Wan Z . Gestational exposure to ambient particulate matter and preterm birth: An updated systematic review and meta-analysis. Environ Res. (2022) 212(Pt C):113381. doi: 10.1016/j.envres.2022.113381 35523275

