# Supplementary material for: Global, regional, and national burden of preterm birth 1990 to 2023, with projections of prevalence to 2050
Source: Front Public Health. 2026 Jul 30;14:1889106. doi: 10.3389/fpubh.2026.1889106 (PMC13469283; doi:10.3389/fpubh.2026.1889106)
Supplement: Supplementary file 1 [file Data_Sheet_1.pdf]

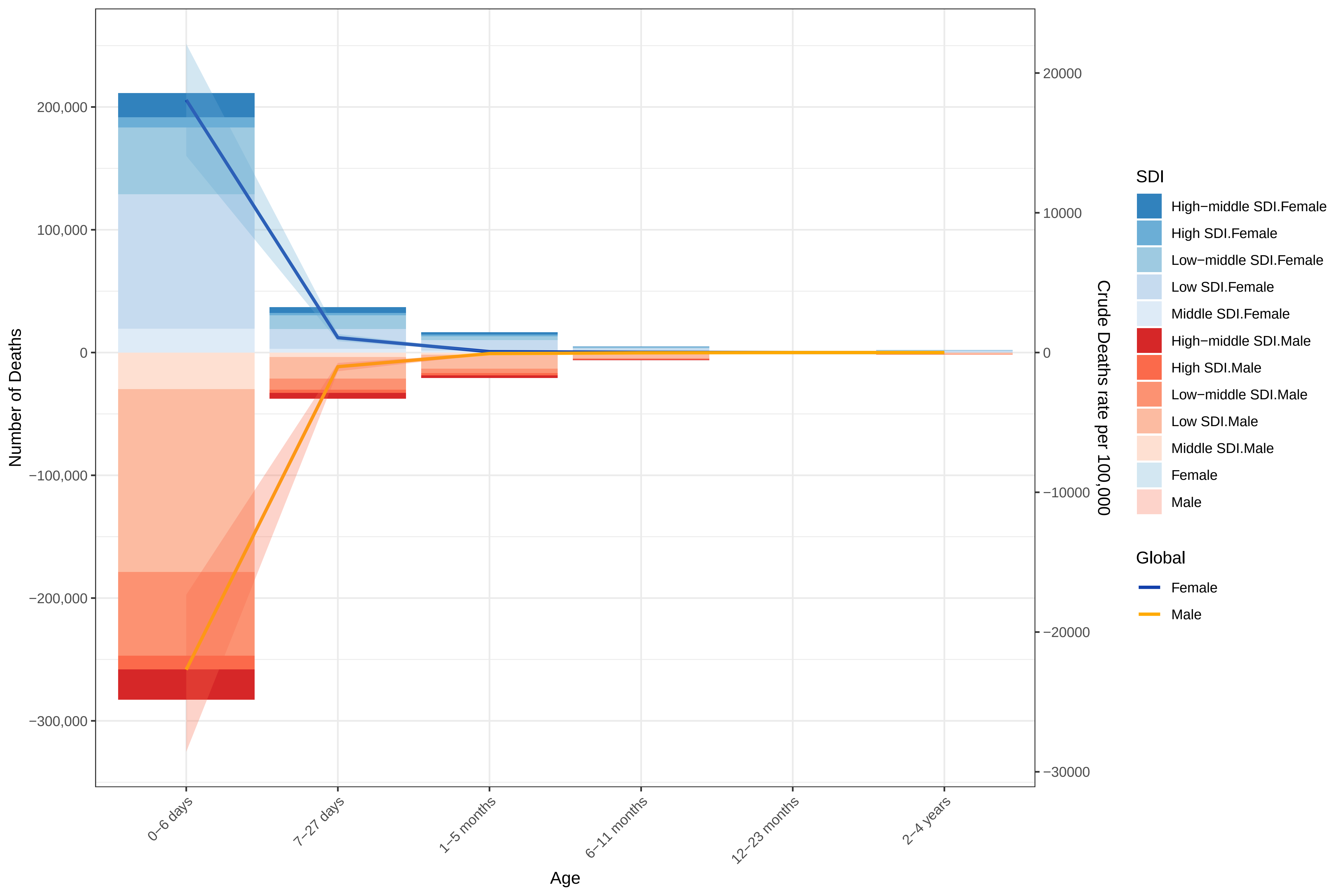
Number of Deaths
200,000
100,000
0
−100,000
−200,000
−300,000
Crude Deaths rate per 100,000
20000
10000
0
−10000
−20000
−30000
0–6 days
7–27 days
1–5 months
6–11 months
12–23 months
2–4 years
Age
SDI
High−middle SDI.Female
High SDI.Female
Low−middle SDI.Female
Low SDI.Female
Middle SDI.Female
High−middle SDI.Male
High SDI.Male
Low−middle SDI.Male
Low SDI.Male
Middle SDI.Male
Female
Male
Global
Female
Male
