# Supplementary material for: Global, regional, and national burden of preterm birth 1990 to 2023, with projections of prevalence to 2050
Source: Front Public Health. 2026 Jul 30;14:1889106. doi: 10.3389/fpubh.2026.1889106 (PMC13469283; doi:10.3389/fpubh.2026.1889106)
Supplement: Supplementary file 2 [file Data_Sheet_2.pdf]

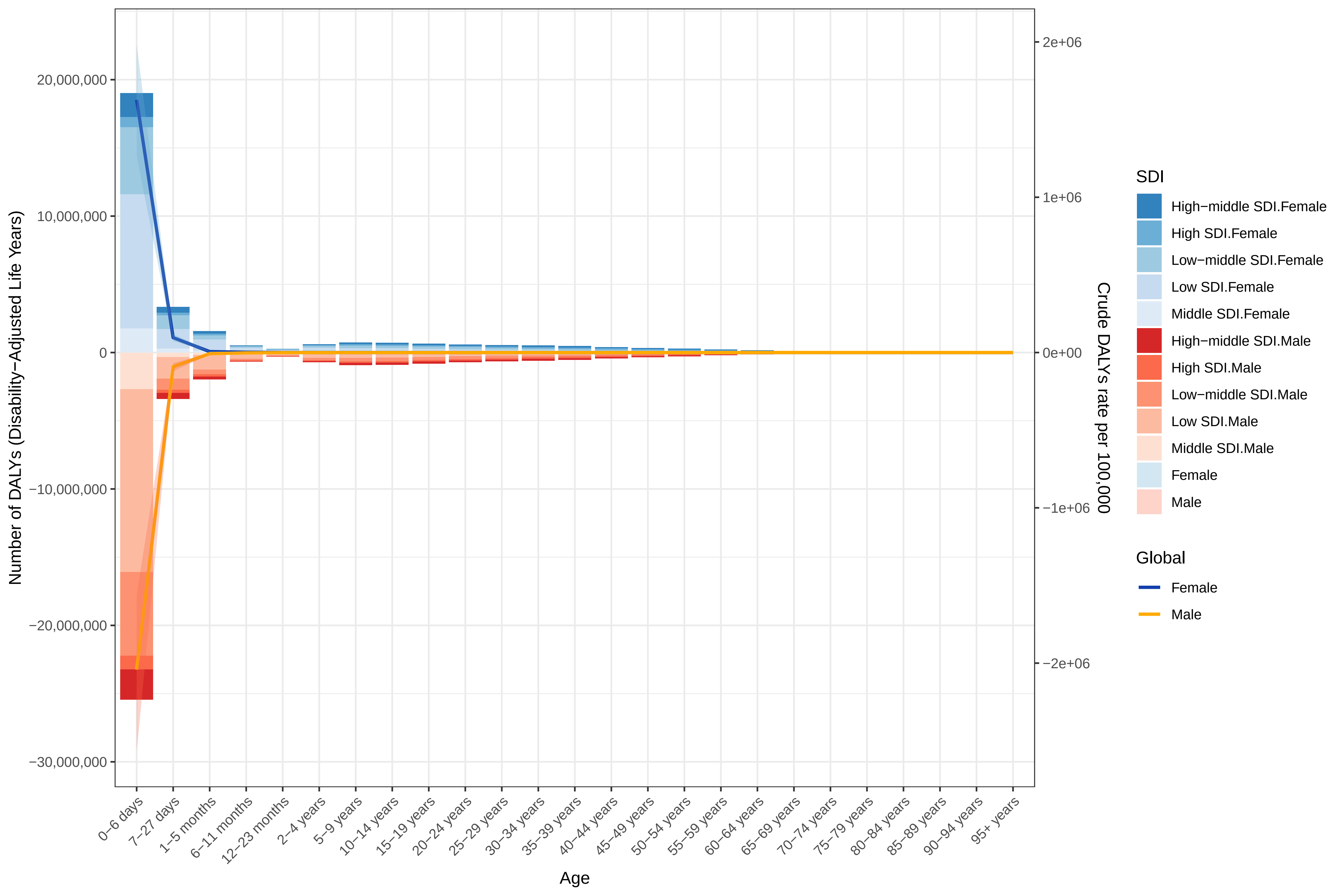

Number of DALYs (Disability−Adjusted Life Years)
20,000,000
10,000,000
0
−10,000,000
−20,000,000
−30,000,000
Crude DALYs rate per 100,000
2e+06
1e+06
0e+00
−1e+06
−2e+06
Age
0−6 days
7−27 days
1−5 months
6−11 months
12−23 months
2−4 years
5−9 years
10−14 years
15−19 years
20−24 years
25−29 years
30−34 years
35−39 years
40−44 years
45−49 years
50−54 years
55−59 years
60−64 years
65−69 years
70−74 years
75−79 years
80−84 years
85−89 years
90−94 years
95+ years
SDI
High−middle SDI.Female
High SDI.Female
Low−middle SDI.Female
Low SDI.Female
Middle SDI.Female
High−middle SDI.Male
High SDI.Male
Low−middle SDI.Male
Low SDI.Male
Middle SDI.Male
Female
Male
Global
Female
Male
