# Supplementary material for: Global, regional, and national burden of preterm birth 1990 to 2023, with projections of prevalence to 2050
Source: Front Public Health. 2026 Jul 30;14:1889106. doi: 10.3389/fpubh.2026.1889106 (PMC13469283; doi:10.3389/fpubh.2026.1889106)
Supplement: Supplementary file 4 [file Data_Sheet_4.pdf]

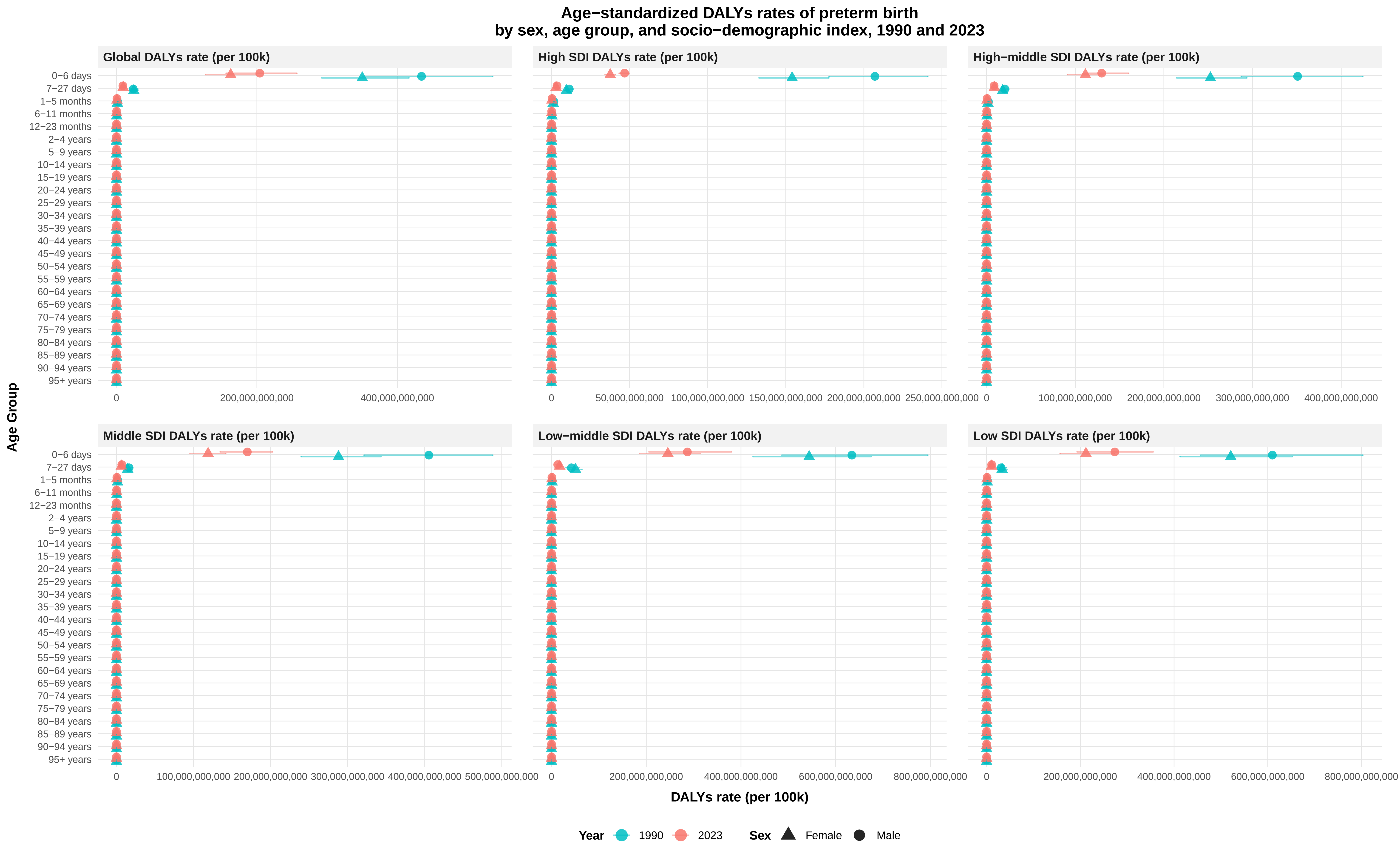

Age−standardized DALYs rates of preterm birth
by sex, age group, and socio−demographic index, 1990 and 2023
Global DALYs rate (per 100k)
High SDI DALYs rate (per 100k)
High−middle SDI DALYs rate (per 100k)
Middle SDI DALYs rate (per 100k)
Low−middle SDI DALYs rate (per 100k)
Low SDI DALYs rate (per 100k)
0−6 days
7−27 days
1−5 months
6−11 months
12−23 months
2−4 years
5−9 years
10−14 years
15−19 years
20−24 years
25−29 years
30−34 years
35−39 years
40−44 years
45−49 years
50−54 years
55−59 years
60−64 years
65−69 years
70−74 years
75−79 years
80−84 years
85−89 years
90−94 years
95+ years
Age Group
0
200,000,000,000
400,000,000,000
50,000,000,000
100,000,000,000
150,000,000,000
200,000,000,000
250,000,000,000
100,000,000,000
200,000,000,000
300,000,000,000
400,000,000,000
100,000,000,000
200,000,000,000
300,000,000,000
400,000,000,000
500,000,000,000
200,000,000,000
400,000,000,000
600,000,000,000
800,000,000,000
DALYs rate (per 100k)
Year
1990
2023
Sex
Female
Male
